# A maximum curvature method for estimating epidemic onset of seasonal influenza in Japan

**DOI:** 10.1186/s12879-019-3777-x

**Published:** 2019-02-20

**Authors:** Jun Cai, Bing Zhang, Bo Xu, Karen Kie Yan Chan, Gerardo Chowell, Huaiyu Tian, Bing Xu

**Affiliations:** 1 Ministry of Education Key Laboratory for Earth System Modeling, Department of Earth System Science, Tsinghua University, Beijing, 100084 China; 2 Joint Center for Global Change Studies, Beijing, 100875 China; 3 School of Public Health (Shenzhen), Sun Yat-sen University, Shenzhen, 518107 China; 4 School of Public Health, Georgia State University, Atlanta, GA 30302 USA; 5 State Key Laboratory of Remote Sensing Science, College of Global Change and Earth System Science, Beijing Normal University, Beijing, 100875 China

**Keywords:** Japan, Influenza surveillance, Epidemic threshold, Non-thresholding method, Segmented regression, Maximum curvature method, MCM

## Abstract

**Background:**

Detecting the onset of influenza epidemic is important for epidemiological surveillance and for investigating the factors driving spatiotemporal transmission patterns. Most approaches define the epidemic onset based on thresholds, which use subjective criteria and are specific to individual surveillance systems.

**Methods:**

We applied the empirical threshold method (ETM), together with two non-thresholding methods, including the maximum curvature method (MCM) that we proposed and the segmented regression method (SRM), to determine onsets of influenza epidemics in each prefecture of Japan, using sentinel surveillance data of influenza-like illness (ILI) from 2012/2013 through 2017/2018. Performance of the MCM and SRM was evaluated, in terms of epidemic onset, end, and duration, with those derived from the ETM using the nationwide epidemic onset indicator of 1.0 ILI case per sentinel per week.

**Results:**

The MCM and SRM yielded complete estimates for each of Japan’s 47 prefectures. In contrast, ETM estimates for Kagoshima during 2012/2013 and for Okinawa during all six influenza seasons, except 2013/2014, were invalid. The MCM showed better agreement in all estimates with the ETM than the SRM (R^2^ = 0.82, *p* < 0.001 vs. R^2^ = 0.34, *p* < 0.001 for epidemic onset; R^2^ = 0.18, *p* < 0.001 vs. R^2^ = 0.05, *p* < 0.001 for epidemic end; R^2^ = 0.28, *p* < 0.001 vs. R^2^ < 0.01, *p* = 0.35 for epidemic duration). Prefecture-specific thresholds for epidemic onset and end were established using the MCM.

**Conclusions:**

The Japanese national epidemic onset threshold is not applicable to all prefectures, particularly Okinawa. The MCM could be used to establish prefecture-specific epidemic thresholds that faithfully characterize influenza activity, serving as useful complements to the influenza surveillance system in Japan.

**Electronic supplementary material:**

The online version of this article (10.1186/s12879-019-3777-x) contains supplementary material, which is available to authorized users.

## Background

Influenza is a common respiratory infectious disease that imposes significant morbidity and mortality impact on public health [1]. Every year, seasonal influenza epidemics are estimated to cause about 3 to 5 million cases of severe illness and up to 650,000 deaths globally [2], placing a substantial burden on health services. To curb these epidemics, the beginning of major influenza activity in each season must be declared. A timely alert of the onset of seasonal influenza epidemic could allow health communities to activate appropriate influenza response plans and prepare for a subsequent dramatic increase in incidence and utilization of health services [3]. In temperate regions such as Japan, seasonal influenza epidemics are expected to occur during winter [4, 5]; however, the exact onset, duration, and severity of these epidemics are not known because of annual differences in the circulating virus strains, population immunity, human mobility, as well as environmental and other factors [6–8]. Therefore, an intuitive and reliable method for estimating epidemic onset is of great interest to public health decision makers because it can help public health agencies to timely respond to the upcoming epidemic peak.

The epidemic onset is technically defined as the time when the incidence exceeds the epidemic threshold [9]. Hence, the algorithm behind the calculation of the epidemic threshold becomes the key to detecting epidemic onset. Without a consensus for calculating epidemic thresholds, a range of approaches with varying complexity have been proposed [6, 8, 10]. The simplest but the most subjective option is to empirically specify a fixed threshold for the epidemic by visual inspection of observations [6, 11–14]. A slightly more quantitative manner of determining a fixed epidemic threshold is to use simple statistics, e.g., mean or median [15–19]. One class of widely used methods for obtaining time-varying epidemic thresholds stem from the periodic regression model proposed by Serfling in 1963 [20]. A variety of Serfling-like regression models have since been developed to detect the onset [15, 21–23] and peak timing [24] of influenza epidemics, and to characterize the seasonal patterns of influenza [25–27]. The Serfling regression model fits the non-epidemic data from previous years and predicts a baseline curve, above which a certain increase is considered the epidemic threshold. However, these Serfling-type approaches have several drawbacks. Firstly, epidemic and non-epidemic periods are required to be predefined based on subjective criteria [28], such as manual removal of epidemic peaks, the proportion of influenza-like illness (ILI) patients among all outpatients (ILI proportion), the proportion of laboratory specimens from ILI patients testing positive for influenza (positive proportion), and so on. The precise determination of epidemic and non-epidemic periods is actually the onset that we would like to estimate. Secondly, the baseline curve is estimated relying on long-term (usually the 5 or more previous years) historical data [13]. Finally, the quantities added to the baseline are varied and not standardized [15, 22].

Several studies have attempted to define epidemic thresholds, taking into account properties of the epidemic curve, e.g., the rate of increase in the number of cases. Nobre and Stroup [29] detected the epidemic onset using the exponential smoothing technique and properties of numerical derivatives of the epidemic curve. This method does not require long-term historical data and can be applied to surveillance series of less than a year; however, prequisites include that the chosen polynomial model must fit the data well, and exploratory analysis is required to choose the parameters of the exponential smoothing model. The World Health Organization (WHO) Regional Office for Europe and the European Center for Disease Prevention and Control have implemented the moving epidemic method (MEM) to determine the baseline influenza activity and epidemic thresholds for influenza surveillance in Europe [8]. The MEM calculates the epidemic start and end after the optimum epidemic duration is firstly found with the slope of the maximum accumulated rates percentage curve less than a predefined criterion δ. Although the MEM can be used for analyzing a single influenza season with as few as 33 weeks of observations, the determination of δ is difficult as it is country-specific. Recently Cheng et al. [30] developed a moving logistic regression method (MLRM) to determine the thresholds of seasonal influenza epidemics across 30 provinces in mainland China. The MLRM approximates the cumulative epidemic curve by a logistic regression model. Following the MEM, the MLRM chooses the optimum epidemic duration with a slight change of R^2^ < 0.01. However, the application of MLRM is limited to symmetric epidemic waves and is not appropriate to asymmetric or bimodal epidemic waves.

While the predominant approaches to detecting epidemic onset are based on thresholds, a few non-thresholding methods have been proposed for estimating epidemic onset. To study the spatiotemporal transmission patterns of influenza, Charu et al. [31] and Geoghegan et al. [7] determined the onset time of epidemics using the segmented regression model (SRM). They fitted a segmented regression model to the first half of the epidemic curve (i.e., the weekly time series of ILI before the peak), where the breakpoint quantifies an abrupt change in incidence and its timing corresponds to the epidemic onset. The SRM does not rely on any threshold and can be applied to a single influenza season without requirements for historical data because it defines epidemic onset totally based on the properties of the epidemic curve.

Charu et al. [31] also demonstrated excellent agreement between influenza epidemic onset estimates derived by the SRM and the Serfling regression model in the United States (US). However, the consistency between epidemic onsets estimated by the SRM and other threshold-based methods using other influenza surveillance systems remains unknown. The lack of reliable information on epidemic onset observations limits the execution of such evaluations. Since 2000, the national epidemic threshold for sentinel surveillance of ILI in Japan has been empirically defined as 1.0 ILI case per sentinel per week (C/S/W) [32, 33]. This epidemic threshold successfully captures a unique feature of the epidemic curve, which means that once the threshold is exceeded, the weekly number of ILI cases increases rapidly and consistently until peaking [34]. Hence, those onsets derived by this empirical threshold method (ETM) for influenza epidemics in Japan can be used as a reference standard for assessing other approaches to estimating epidemic onsets.

The thresholds for the onset and end of influenza epidemic are supposed to vary across Japanese prefectures [35]. Yet, no appropriate epidemic threshold exists for each prefecture. We propose a novel statistical method, the maximum curvature method (MCM), to determine prefecture-specific onsets of influenza epidemics in Japan. This method is based on the maximum curvature of the epidemic curve, which makes the best use of the epidemic curve’s unique feature and retains the advantages of non-thresholding methods for estimating epidemic onset. As we focus on the non-thresholding methods, in this study, epidemic onset estimates derived by both the MCM and SRM are evaluated in comparison with the reference epidemic onsets obtained by the ETM with a fixed value of 1.0 C/S/W. Finally, prefecture-specific thresholds for epidemic onset and end are established using the MCM.

## Methods

### Study area and ILI surveillance data

Japan is a bow-shaped strip of islands, stretching from 24°N to 46°N for approximately 2400 km. At its widest point, Japan is no more than 230 km across. Japan is divided into 47 prefectures for local administration. Hokkaido is the northernmost prefecture; Okinawa is the southernmost prefecture. Most regions of Japan lie in the temperate zone with humid subtropical climate. However, Japan’s climate varies from a cool humid continental climate in the north, such as in northern Hokkaido, to a warm tropical rainforest climate in the south, such as in Ishigaki, Okinawa.

Influenza (excluding avian influenza and pandemic influenza, e.g. novel influenza or re-emerging influenza) is subject to sentinel surveillance under the National Epidemiological Surveillance for Infectious Disease in Japan. The number of patients diagnosed with ILI is reported from approximately 5000 sentinel medical institutions (SMIs) (3000 for pediatrics and 2000 for internal medicine) across Japan on a weekly basis (ISO 8601 week date system according to the Weeks Ending Log [36]). The criteria for reporting ILI used by SMIs have been previously described elsewhere [37]. The data are aggregated at the National Institute of Infectious Diseases into weekly total number of cases and weekly average number of cases per sentinel for both the national and prefectural levels [37]. The surveillance data tables are published on the website of the Infectious Disease Weekly Report (IDWR) [38] every Tuesday. A detailed description of infectious diseases surveillance system in Japan has been made available [39].

In our study, an influenza season was defined to range anywhere from week 35 in September of each year up to week 34 in August of the following year. We downloaded IDWR surveillance data tables from week 35 of 2012 to week 34 of 2018 (from 2012-09-02 to 2018-08-26 in terms of week ending date). Our study period covered six influenza seasons from 2012/2013 through 2017/2018 (Additional file 1: Fig. S1). Only the weekly number of ILI cases per sentinel was used in the following estimation of epidemic onsets, so as to be compatible with the empirical epidemic threshold.

### Methods for estimating epidemic onset

We estimated the onset time of influenza epidemics in each prefecture for each of the six influenza seasons from 2012/2013 to 2017/2018 using three methods: the ETM, SRM, and MCM. The epidemic end is equivalent to the epidemic onset in reverse chronological order. The duration of an epidemic is defined as the period from its onset time to its ending time. Therefore, we focused on describing the algorithm for estimating epidemic onset.

#### The empirical threshold method (ETM)

The ETM defines an epidemic as occurring when the weekly number of ILI cases per sentinel has been reported to exceed a prespecified threshold *Y*_0_ for three consecutive weeks [40]. The first week of the three consecutive weeks corresponds to the epidemic onset. We used the criterion *Y*_0_ = 1.0 C/S/W, which is the threshold for the nationwide onset of an influenza epidemic in Japan. This threshold was empirically defined in the year 2000 based on more than 10 years of observations from sentinel surveillance of influenza in Japan [34]. The details of implementing the ETM are described in the Additional file 1: Text S1 and Fig. S2.

#### The segmented regression method (SRM)

Different from the above threshold-based method, the SRM fits piecewise linear models to determine the breakpoint in the first half of the epidemic curve, which corresponds to the epidemic onset. In other words, the breakpoint is the optimal knot location with the maximal difference-in-slope between the two fitted straight lines (Additional file 1: Figure S3). To find the optimal breakpoint, the log-likelihood function for the breakpoint is maximized. Further details of using the SRM to determine epidemic onset refer to [7, 31]. We implemented the SRM using the R package segmented [41], and the procedure is summarized in the Additional file 1: Text S2. An illustration of the SRM is shown in Additional file 1: Figure S3.

#### The maximum curvature method (MCM)

Given the unique feature of the epidemic curve in Japan, it may be more appropriate to identify the epidemic onset in terms of curvature. Therefore, we developed the MCM to detect epidemic onset and end. Inspired by the SRM definition of epidemic onset as the point of maximum change in the slope, the MCM defines epidemic onset as the point of maximum curvature located within the increasing phase of the epidemic curve. Likewise, epidemic end is defined as the point of maximum curvature located within the decreasing phase of the epidemic curve. To reduce the effect of small fluctuations in the epidemic curve, instead of directly calculating the osculating circle at each point on the curve, the MCM fits a least-squares circle to the *n* points around it. *n* ≥ 3 because three points are required to determine a circle and *n* is odd for the sake of symmetry. The curvature of the fitted circle only measures how fast the epidemic curve is changing direction at a given point. We further used the directional angle of the tangent vector at the given point to indicate its changing direction. In the first half of the epidemic curve, the point with maximum curvature and a directional angle between [0°, 90°] is defined as the epidemic onset; in the second half, the point with maximum curvature and a directional angle between [270°, 360°] is determined as the epidemic end. Any possible points that occur above an upper threshold, *h* C/S/W, are eliminated, because they are already in an epidemic state.

Let {*y*_*t*_, *t* = 1, 2,  … , *T*} denote the weekly epidemic curve of an influenza season with *T* weeks, where *y*_*t*_ is the number of ILI cases per sentinel reported at week *t*, which is referred to as intensity hereafter, for the sake of simplicity. The steps for using the MCM to detect epidemic onset and end are as follows.

Step 1. At a given point *K* (*t*, *y*_*t*_)(*t* = 1, 2,  … , *T*), a circle with center $O\ \left({t}_{\mathrm{c}},{y}_{t_{\mathrm{c}}}\right)$ and radius *r* is determined by least-squares fitting to *n* points $\left(t-\frac{n-1}{2},{y}_{t-\frac{n-1}{2}}\right),\dots, \left(t+\frac{n-1}{2},{y}_{t+\frac{n-1}{2}}\right)$ surrounding *K*, using the algorithm proposed by Pratt [42]. When *K* is at the edge of the epidemic curve ($t=1,\dots, \frac{n-1}{2}\ \mathrm{or}\ t=T-\frac{n-3}{2},\dots, T$), the first (or last) two points of the epidemic curve are linearly extrapolated to pad the curve with $\frac{n-1}{2}$ extra points. The raw curvature *C*_*t*_ at *K* is the reciprocal of the radius *r*.

Step 2. The tangent point $P\ \left(\widehat{t},\widehat{y_t}\right)$ closest to *K*, is determined by intersecting the line *OK* with the fitted circle. The directional angle *θ*_*t*_ (in degrees) of the tangent vector $\overrightarrow{PQ}$ is then calculated.

Step 3. The raw curvature *C*_*t*_ is filtered based on the directional angle *θ*_*t*_ and the upper threshold *h*.

$$ {C}_t^{\prime }=\left\{\begin{array}{c}{C}_tI\left({0}^{{}^{\circ}}\le {\theta}_t\le {90}^{{}^{\circ}}\right)I\left({y}_t\le h\right),\mathrm{if}\ t\le {t}_p,\\ {}{C}_tI\left({270}^{{}^{\circ}}\le {\theta}_t\le {360}^{{}^{\circ}}\right)I\left({y}_t\le h\right),\mathrm{otherwise}\end{array}\right. $$

where *I* is an indicator function, ${t}_p=\underset{t=1,\dots, T}{\arg \max}\left\{{y}_t\right\}$ is the peak timing.

Step 4. Find the points with the maximum filtered curvature ${t}_o=\underset{t=1,\dots, {t}_p}{\arg \max}\left\{{C}_t^{\prime}\right\}$ and ${t}_e=\underset{t={t}_p,\dots, T}{\arg \max}\left\{{C}_t^{\prime}\right\}$ for each half of the epidemic curve.

Step 5. The coordinates of the tangent point at $\left(\widehat{t_o},\widehat{y_{t_o}}\right)$ correspond to the epidemic onset and the epidemic onset intensity. Likewise, the coordinates of the tangent point at $\left(\widehat{t_e},\widehat{y_{t_e}}\right)$ correspond to the epidemic end and the epidemic ending intensity.

**Fig. 1:**
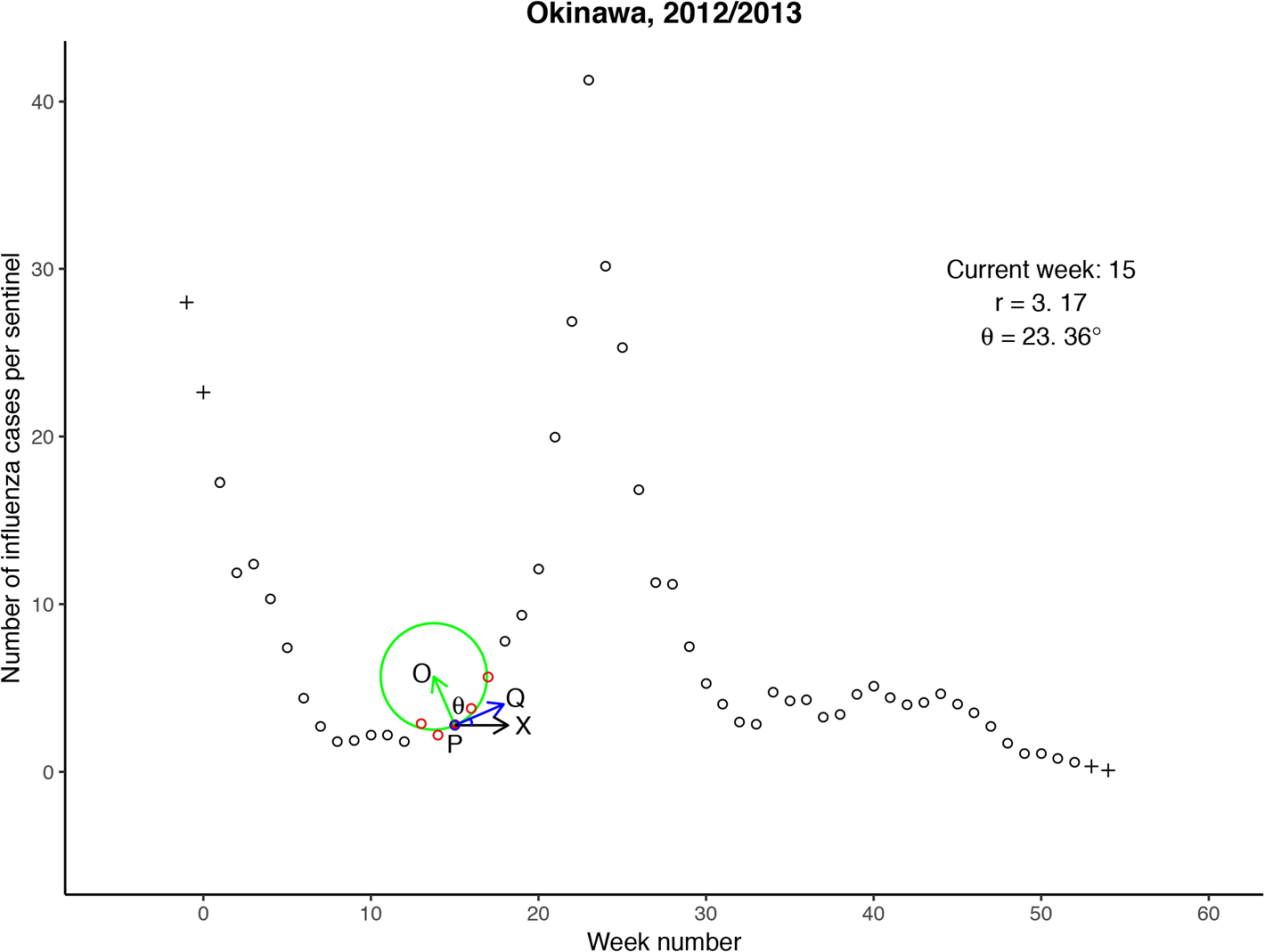
Illustration of least-squares circle fitting at epidemic onset week for Okinawa during 2012/2013. The five red (both solid and empty) points are used for fitting a least-squares circle at current week 15 (solid red point). The plus symbols are two padded points at the edge of the epidemic curve by linearly extrapolating the first (or last) two points. The fitted circle is depicted by the green curve, and its radius *r* is shown in the top right annotation. The arrows respectively represent the positive x-axis vector $\overrightarrow{PX}$ (in black), the tangent vector $\overrightarrow{PQ}$ (in blue), and the normal vector $\overrightarrow{PO}$ (in green) at the tangent point *P*. *θ* denotes the directional angle of $\overrightarrow{PQ}$, and its value is also shown in the top right annotation

**Fig. 2:**
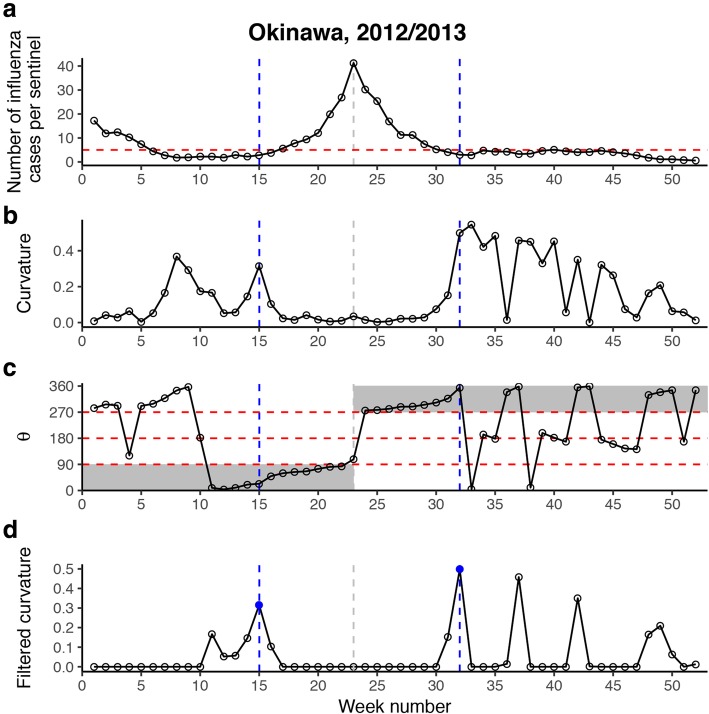
Illustration of the maximum curvature method (MCM). Panel **a** demonstrates the epidemic curve of weekly number of ILI cases per sentinel for Okinawa during 2012/2013. The red dashed horizontal line indicates the upper threshold of *h*=5.0 ILI cases per sentinel per week. Panel **b** shows the raw curvature of the fitted least-squares circle in each week. Panel **c** shows the directional angle θ of the tangent vector $\overrightarrow{PQ}$ in each week. The red dashed horizontal lines indicate 90°, 180°, and 270°. The gray shaded areas respectively represent [0°, 90°] during the first half of the epidemic curve, and [270°, 360°] during the second half, within which *θ* would be expected to lie. Panel **d** shows the curvature filtered by *h* and *θ* in each week. The blue solid points represent the maximum filtered curvatures in the first and second halves of the epidemic curve. The corresponding blue dashed vertical lines across the panels indicate the epidemic onset and ending weeks

In our study, *n* = 5 and *h* = 5.0 were used for estimating epidemic onsets, ends, and their intensities. The MCM is illustrated in Figs. 1 and 2 with an animation of fitting least-squares circles provided in Additional file 2: Movie S1.

### Comparison of epidemic characteristic parameters derived by different methods

For each season, epidemic characteristic parameters including epidemic onset, end, duration, and intensities at epidemic onset and end were estimated using the above ETM, SRM, and MCM, nationally and for each prefecture. The threshold for the nationwide onset of an influenza epidemic in Japan has been empirically defined as 1.0 C/S/W since 2000 [34]. However, the prefecture-specific thresholds for epidemic onsets have yet to be determined. We presumed that the epidemic onset thresholds at prefecture level would be similar to the national threshold and thus specified *Y*_0_ to be 1.0 C/S/W when using the ETM to estimate epidemic characteristic parameters for each prefecture. Owing to the continued success of the nationwide epidemic onset indicator in Japan, estimates of the ETM using this indicator were used as the reference standard, against which epidemic characteristic parameter estimates using the other two methods were compared. A sensitivity analysis varying *n* (3, 5, and 7) and *h* (4.0, 6.0, 8.0, and 10.0) was performed to examine the MCM’s robustness. For each combination of *n* and *h*, epidemic characteristic parameters estimated by the MCM were also compared with those from the ETM.

### Establishment of prefecture-specific thresholds for epidemic onset and end

With the epidemic characteristic parameters estimated by the MCM (*n* = 5, *h* = 5.0) in hand, the prefecture-specific thresholds for epidemic onset were calculated by averaging the epidemic onset intensities over the six available seasons, 2012/2013 to 2017/2018. The prefecture-specific epidemic ending thresholds were also calculated using the same procedure.

All methods and analyses were implemented in R 3.4.2 [43]. The datasets and codes are available under MIT license at the GitHub repository [44].

## Results

### Descriptive statistics of epidemic characteristic parameter estimates

The epidemic characteristic parameter estimates using the ETM, SRM, and MCM for each of the 47 prefectures from 2012/2013 to 2017/2018 are summarized in Table 1 and Additional file 1: Figure S4. Across the six seasons, epidemic onsets estimated by the SRM (mean 18.2 weeks) were much later than those derived from the ETM (mean 15.2 weeks); epidemic ends from the SRM (mean 30.7 weeks) were considerably earlier than those derived from the ETM (mean 37.1 weeks). The resultant epidemic durations estimated by the SRM (mean 13.5 weeks) were notably shorter than those estimated by the ETM (mean 22.7 weeks). Furthermore, epidemic onset and ending intensities estimated by the SRM (mean 5.72 and 6.90, respectively) were much higher than the empirical threshold of 1.0 C/S/W. By contrast, epidemic characteristic parameters estimated by the MCM (mean 15.0, 35.5, and 21.5 weeks for epidemic onset, end, and duration, respectively) were very close to those derived by the ETM, particularly epidemic onset and ending intensities (mean 0.78 and 1.40, respectively). It is noted that the interquartile ranges (IQRs) of the epidemic ending intensities derived by the MCM during seasons 2012/2013 (mean 1.99, IQR 1.60), 2014/2015 (mean 1.96, IQR 1.52), and 2016/2017 (mean 1.67, IQR 1.74) were larger than those during the other three seasons (mean 1.07, IQR 0.57 for 2013/2014; mean 0.66, IQR 0.49 for 2015/2016; mean 1.01, IQR 0.43 for 2017/2018) (Additional file 1: Figure S4). Furthermore, the dominant influenza virus subtypes in these three seasons were all A(H3) (Table 1).

**Table 1:** Summary statistics of epidemic characteristic parameters estimated by the ETM, SRM, and MCM from 2012/2013 to 2017/2018

Parameters	Methods	2012/2013	2013/2014	2014/2015	2015/2016	2016/2017	2017/2018	Mean
Onset^a^ (weeks)	ETM	16.4	17.0	13.9	19.3	12.2	12.6	15.2
	SRM	18.9	19.2	15.8	20.6	17.9	17.1	18.2
	MCM	16.0	17.0	13.2	18.5	12.7	12.4	15.0
End (weeks)	ETM	38.7	37.7	36.5	37.6	37.7	34.3	37.1
	SRM	29.4	34.6	26.5	34.4	30.1	29.4	30.7
	MCM	34.3	37.5	32.4	38.1	36.3	34.1	35.5
Duration (weeks)	ETM	23.0	21.8	23.5	19.1	26.3	22.6	22.7
	SRM	11.5	16.3	11.7	14.8	13.3	13.3	13.5
	MCM	19.3	21.6	20.2	20.6	24.6	22.7	21.5
Onset intensity^b^	ETM	1	1	1	1	1	1	1
	SRM	4.25	3.90	5.08	3.30	7.95	9.87	5.72
	MCM	0.70	0.87	0.50	0.61	1.13	0.84	0.78
Ending intensity	ETM	1	1	1	1	1	1	1
	SRM	7.39	4.04	8.74	5.13	8.05	8.06	6.90
	MCM	1.99	1.07	1.96	0.66	1.67	1.01	1.40
Dominant subtype^c^		A(H3)	A(H1N1)pdm09	A(H3)	A(H1N1)pdm09	A(H3)	B/Yamagata	–

^a^Epidemic onset and end are weeks since week 34 of each year

^b^Epidemic onset and ending intensities represent the weekly number of ILI cases per sentinel at epidemic onset and end, respectively

^c^Information on dominant subtype for each influenza season are from [45–50]

It is noteworthy that valid epidemic characteristic parameters were obtained when applying the SRM or MCM to all 47 prefectures during all six seasons, but the ETM failed to produce results in a few prefectures located in the southern part of Japan for several seasons (Table 2). In Okinawa, 2013/2014 was the only season in which the ETM produced valid estimates among all five epidemic parameters. The ETM also returned an invalid epidemic onset for Kagoshima during 2012/2013.

**Table 2:** Prefectures with invalid epidemic characteristic parameters estimated by the ETM

Season	Onset	End	Duration	Onset intensity	Ending intensity
2012/2013	Kagoshima Okinawa		Kagoshima Okinawa	Kagoshima Okinawa	
2013/2014					
2014/2015		Okinawa	Okinawa		Okinawa
2015/2016	Okinawa		Okinawa	Okinawa	
2016/2017		Okinawa	Okinawa		Okinawa
2017/2018	Okinawa	Okinawa	Okinawa	Okinawa	Okinawa

### Agreement between the SRM, MCM and ETM on epidemic onset, end, and duration estimates

**Fig. 3:**
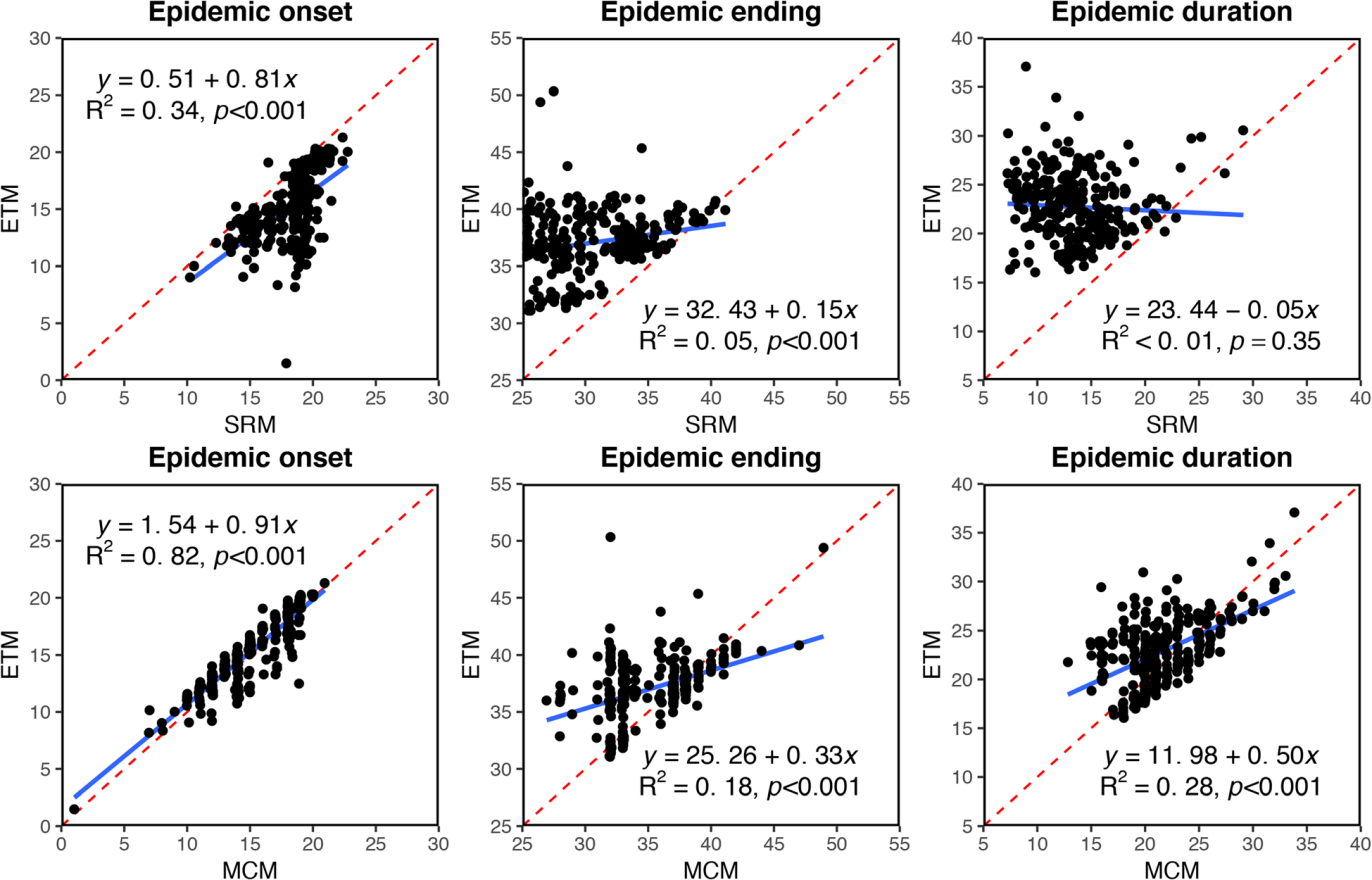
Agreement between epidemic characteristic parameter estimates by comparing the SRM and MCM with the ETM. The regression equation, R^2^ and *p* value are annotated in each plot. The regression line is shown as the blue solid line in each plot. The 1:1 lines are given as the red dashed lines for reference

Epidemic onset, end, and duration estimates derived from the SRM and MCM were respectively compared with those estimated by the ETM using linear regression. The results indicate that regardless of the epidemic characteristic parameters, the agreement between the MCM and ETM was much better than that between the SRM and ETM (Fig. 3). Compared with the ETM, the SRM generally overestimated epidemic onset, with moderate agreement (slope = 0.81, R^2^ = 0.34, *p* < 0.001), whereas it underestimated epidemic end, with poor agreement (slope = 0.15, R^2^ = 0.05, *p* < 0.001). These results led to insignificant agreement between epidemic durations derived by the SRM and ETM (slope =  − 0.05, R^2^ < 0.01, *p* = 0.35). In contrast, epidemic onset estimates derived by the MCM showed good consistency with those from the ETM (slope = 0.91, R^2^ = 0.82, *p* < 0.001). Like the SRM, the MCM also tended to underestimate epidemic end, but with better agreement (slope = 0.33, R^2^ = 0.18, *p* < 0.001). Moderately significant agreement (slope = 0.50, R^2^ = 0.28, *p* < 0.001) was observed between epidemic duration estimates derived by the MCM and ETM.

To explore the robustness of MCM, the agreement of epidemic characteristic parameters determined by the ETM and MCM using different *n* and *h* were further assessed (Additional file 1: Figures. S6-S8). The sensitivity analysis results were summarized in Table 3. For all combinations of *n* and *h*, the agreement of epidemic onset was the best among the three epidemic characteristic parameters. With a fixed *h*, *n *= 5 and 7 had about the same agreement of epidemic onset and duration, which were much better than *n *= 3. By contrast, the agreement of epidemic end was relatively robust to *n*. With a fixed *n*, the agreement of epidemic onset was robust to *h *= 4.0, 6.0, and 8.0, but decreased when *h *= 10.0. The agreement of epidemic end decreased slightly when *h *≤ 8.0, but was robust to *h *> 8.0. The agreement of epidemic duration decreased with the increase of *h*. In short, the epidemic characteristic parameters, particularly the epidemic onset, determined by the MCM were relatively robust when *n *= 5 or 7 and *h *= 4.0, 6.0, or 8.0.

**Table 3:** Agreement of epidemic characteristic parameters determined by the ETM and MCM using different *n* and *h*

Parameters	Onset			End			Duration		
	*n* = 3	*n* = 5	*n* = 7	*n* = 3	*n* = 5	*n* = 7	*n* = 3	*n* = 5	*n* = 7
*h* = 4.0	0.43^a^	0.81	0.82	0.20	0.19	0.14	0.17	0.29	0.27
*h* = 6.0	0.41	0.81	0.80	0.13	0.16	0.13	0.10	0.24	0.24
*h* = 8.0	0.41	0.81	0.80	0.11	0.10	0.08	0.09	0.18	0.18
*h* = 10.0	0.35	0.71	0.68	0.10	0.11	0.08	0.07	0.14	0.15

^a^The coefficient of determination (R^2^) of linear regression model used for comparison is reported

### Prefecture-specific epidemic onset and ending thresholds

**Fig. 4:**
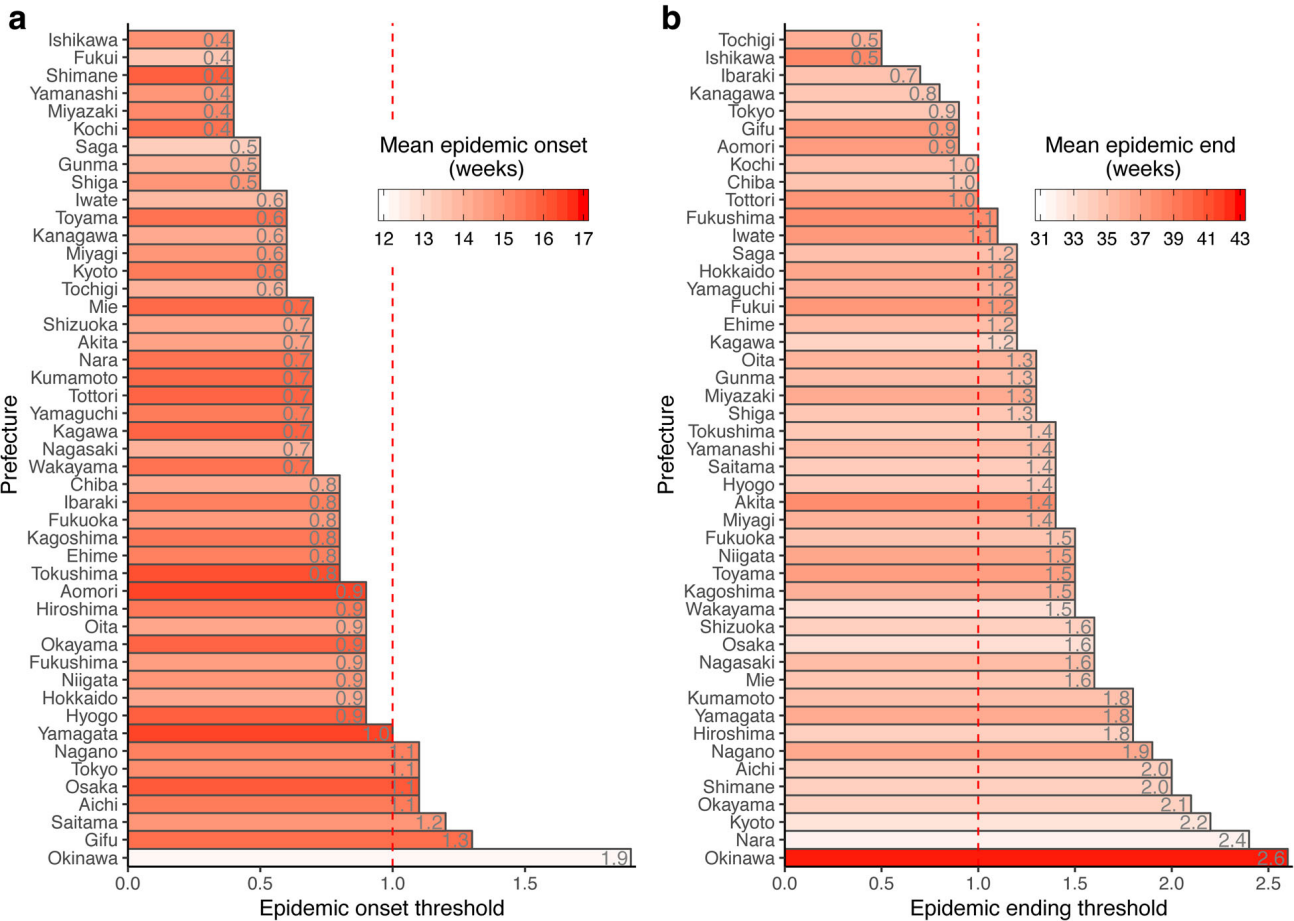
Epidemic (Panel **a**) onset and (Panel **b**) ending thresholds (rounded to one decimal place) established for the 47 prefectures in Japan using the MCM with *n *= 5 and *h *= 5.0. The relative ordering of epidemic onsets (ends) is depicted by the bar color: white indicates prefectures with the earliest epidemic onsets (ends), and red indicates prefectures with the latest epidemic onsets (ends). The prefectures are sorted by increasing epidemic onset (ending) thresholds

The epidemic onset and ending thresholds established using the MCM with *n *= 5 and *h *= 5.0 showed variability across prefectures (Fig. 4). The epidemic onset thresholds ranged from 0.4 C/S/W for Ishikawa to 1.9 C/S/W for Okinawa, whereas the epidemic ending thresholds ranged from 0.5 C/S/W for Tochigi to 2.6 C/S/W for Okinawa. What stands out is that Okinawa, the southernmost prefecture located in the subtropics, had the largest epidemic onset and ending thresholds, while its mean epidemic onset was the earliest (12.2 weeks) and its mean epidemic end was the latest (42.7 weeks). Most prefectures (39/47) had an epidemic onset threshold below the current nationwide epidemic onset indicator of 1.0 C/S/W. In contrast, most prefectures (37/47) had an epidemic ending threshold above the indicator (Fig. 4). In addition, the epidemic onset and ending thresholds showed a statistically significant correlation (*r* = 0.34, *p *= 0.02).

## Discussion

In this study, three methods including the ETM, SRM, and MCM, were used to estimate epidemic characteristic parameters for each of the 47 prefectures in Japan during each of the six influenza seasons from 2012/2013 to 2017/2018. Among them, the ETM is a thresholding method to detect epidemic onset based on the nationwide epidemic onset threshold of 1.0 C/S/W. The SRM is an existing non-thresholding method for capturing the breakpoint of the epidemic curve as the epidemic onset. The MCM is also a non-thresholding method that we proposed to detect epidemic onset based on the maximum curvature of the epidemic curve. Proper evaluations of methods for detecting epidemic onset are often impaired because of a lack of suitable datasets with reliable information on the occurrence of epidemics [29]. To address this issue, in the present study, estimates from the ETM were used as reference standards to evaluate the performance of the other two methods.

The incompleteness of ETM estimates suggests that the empirical epidemic threshold is not appropriate for the levels of influenza activity observed in prefectures located at or near the southernmost part of Japan, such as Okinawa and Kagoshima (Table 2). The severe lack of valid ETM estimates in Okinawa resulted from a level of background influenza activity that was higher than the empirical epidemic threshold of 1.0 C/S/W. It has been recognized that background influenza activity is high throughout the year in tropical regions [51]. Hence, the influenza seasonality is less defined in Okinawa, where the lowest influenza activity usually occurs later than in other, more northern prefectures (Additional file 1: Figure S5). By contrast, the epidemic onset and ending thresholds (1.9 and 2.6 C/S/W) for Okinawa established using the proposed MCM were the largest, and much higher than those of other prefectures and the empirical epidemic threshold of 1.0 C/S/W (Fig. 4), faithfully reflecting the characteristics of influenza epidemics in Okinawa.

The epidemic curves in all prefectures were asymmetrical because when approaching the epidemic end, the second half of the epidemic curve was relatively gentle compared with the first half, as demonstrated in the 2014/2015 season (Additional file 1: Figure S5). This asymmetry of the epidemic curve not only explains why better agreement with the ETM was achieved for epidemic onset than for epidemic end, regardless of the method used, but also suggests that thresholds for epidemic onset and end are likely to be different and should be established individually. The high consistency between the MCM and ETM guarantees the continuity of using epidemic thresholds derived by the MCM in the Japanese sentinel surveillance system for influenza. Although the prefecture-specific thresholds for epidemic onset and end were established using the only six available influenza seasons, these thresholds can be further refined as more data become available in the future. In addition to the mean statistic used in the present study, other procedures for calculating the thresholds [8] are worth exploring.

The IQRs of the epidemic ending intensities derived by the MCM during 2012/2013, 2014/2015, and 2016/2017 were wider than those during the other three seasons (Additional file 1: Figure S4). This may be explained by the severity of epidemics. In Japan, the 2012/2013, 2014/2015, and 2016/2017 influenza seasons were characterized by the predominance of the A(H3) subtype whereas the dominant virus subtypes in the other three seasons were A(H1N1)pdm09 and B/Yamagata. Seasonal influenza epidemics dominated by A(H3N2) subtype are generally more severe than those dominated by A(H1N1) and B [52], which may affect the shape of the epidemic curve. Therefore, establishment of epidemic thresholds, particularly the epidemic ending thresholds, could incorporate information on the dominant influenza virus subtype.

The proposed MCM has several properties that make it broadly applicable for estimating epidemic onset in public health surveillance. First, the MCM is intuitive as it defines epidemic onset by capturing the local point with maximum curvature. The MCM is a non-thresholding approach to determining epidemic onset that is based entirely on the shape of the epidemic curve. During implementation of the MCM, an upper threshold *h* is prespecified to limit the search scope for points. However, the sensitivity analysis suggests that the MCM is robust to *h* for a wide range (Table 3). Therefore, this threshold is not required to be as precise as *Y*_0_ in the ETM, and is easy to be set. Moreover, it also provides the flexibility to adjust the search scope for points according to the background levels of influenza activity. These properties together with the success of Okinawa give the MCM the potential to estimate epidemic characteristic parameters in the subtropics and tropics where various respiratory pathogens that can cause acute respiratory illness, such as respiratory syncytial virus, parainfluenza virus etc., circulate year round [18]. Consequently, the patterns of influenza in subtropical and tropical regions are complex with year-round high background rate of acute respiratory illness [51] and lack of apparent ILI seasonality [18]. The recent experience of establishing influenza epidemic thresholds in Cambodia using the WHO method [19] suggests that unlike in temperate regions, the ILI syndromic surveillance data was less useful for setting thresholds [18]. Therefore, priority to virological surveillance data, such as the positive proportion [30], the product of the ILI proportion and the positive proportion, should be given when applying the MCM to establish thresholds for influenza epidemics in subtropical and tropical regions.

Second, in contrast to the widely used Serfling-like regression models requiring long series of historical data to estimate model parameters [13, 20, 22, 26], parameters of the MCM are prespecified. This means the MCM can be applied in areas with limited historical data and in analyzing influenza pandemics that usually last for a single season. Epidemic onsets determined using empirical thresholds [12], Serfling-type regression model [21], and the SRM [7, 31] have been used to investigate spatial transmission of both influenza pandemics and epidemics. New insights into the spatial transmission of influenza may be gained using the MCM as it defines epidemic onset totally based on the properties of the epidemic curve.

Third, although the calculation in the MCM is more complex than that in the SRM, the estimates derived using our novel MCM were in much better agreement with those derived using the ETM. The high consistency between epidemic onsets derived by the ETM and MCM implies that curve properties, such as the curvature, may have been taken into consideration during the determination of the national epidemic onset indicator in Japan. A comparison conducted by Charu et al. [31] showed excellent agreement between estimates of influenza epidemic onset in the US derived by the SRM and Serfling-like regression method, which in essence determines epidemic onset based on thresholds. In constrast, the agreement between the ETM and SRM was poor in Japan. This may be linked to the differences in sentinel surveillance systems for influenza in the US and Japan.

Finally, the MCM is robust not only to model parameters *n* and *h* but also to the partitioning of the influenza seasons and the determination of the epidemic peak. Regarding the estimation of epidemic onset, the MCM calculates the curvature at each point by fitting a least-square circle using only *n* points around the current one. While searching for the local point of maximum curvature, the MCM also takes into account the changing direction of the curvature at each point, which ensures that only points in the ascending phase of the epidemic curve are targeted. In contrast, the SRM fits two broken lines, using all points in the first half of the epidemic curve. Therefore, when the influenza season begins and ends could have an impact on the epidemic onset estimate. In the present study, it was appropriate to define the start of each influenza season as week 35 with the exception of Okinawa during 2012/2013, 2014/2015, and 2016/2017 (Additional file 1: Figure S3 and S5). For example, during 2012/2013 in Okinawa, the influenza season should have been defined to start around week 44. The first broken line fitted by the SRM included approximately the last 10 weeks of the previous influenza season, which resulted in a biased epidemic onset estimate toward earlier weeks. In this case, the curvatures for these weeks is filtered out by the MCM as their directional angles were not between [0°, 90°] (Fig. 2C and D). Furthermore, taking the direction of curvature into consideration may enable the MCM to overcome the constraint of the MLRM [30] and to be applicable to multiple epidemic waves of influenza observed in subtropical and tropical regions, such as southern China [25]. In addition, the SRM is more sensitive to the determination of the epidemic peak timing than the MCM. However, epidemic peaks may suffer from large fluctuations, such as the sharp decrease in ILI activity during the National Day Holiday in the 2009 pandemic in China [53]. Under such circumstances, the SRM will result in a large bias in the epidemic onset estimates.

There are several limitations to the proposed MCM that deserve consideration. First, the MCM can only be used in retrospective analysis of epidemics because data from later weeks are required for fitting the least-square circles. Second, the MCM implicitly relies on the smoothness of the epidemic curve. For epidemic curves with small fluctuations, we can address this limitation by increasing the number of points (e.g., *n* = 7) used for fitting least-square circles. For irregular epidemic curves with large and frequent fluctuations, techniques such as Savitzky-Golay filtering [54], among others, may be used to smooth the epidemic curve before applying the MCM. Finally, in comparison with the SRM, the MCM cannot provide confidence intervals for epidemic onset estimates, which limits the ability of the MCM to take uncertainties into account.

## Conclusions

In conclusion, our findings indicate that the nationwide epidemic onset threshold of 1.0 C/S/W currently used in the sentinel system for influenza surveillance in Japan should be adjusted for each prefecture, especially for Okinawa. The proposed MCM shows better agreement with the ETM than the SRM and performs very well in the context of Japanese influenza surveillance. The prefecture-specific thresholds for epidemic onset and end established using the MCM could serve as useful complements to the influenza surveillance system in Japan. Further research should be undertaken to evaluate the applicability of the MCM in different public health surveillance systems or in tropical and subtropical zones, and in detecting the onset of influenza pandemics.

## Additional files

Additional file 1: **Text S1.** Implementation of the empirical threshold method (ETM). **Text S2.** Implementation of the segmented regression method (SRM). **Figure S1.** Epidemic curves of weekly number of influenza cases per sentinel for the 47 prefectures in Japan from 2012-09-02 to 2018-08-26. **Figure S2.** Illustration of the empirical threshold method (ETM). **Figure S3.** Illustration of the segmented regression method (SRM). **Figure S4.** Box plots of epidemic characteristic parameters estimated by the ETM, SRM, and MCM for the 47 prefectures in Japan from 2012/2013 to 2017/2018. **Figure S5.** Epidemic onset and end estimates in Japan and three representative prefectures during influenza seasons 2012/2013–2017/2018. **Figure S6.** Sensitivity analysis of different *n* and *h* for epidemic onset. **Figure S7.** Sensitivity analysis of different *n* and *h* for epidemic end. **Figure S8.** Sensitivity analysis of different *n* and *h* for epidemic duration. (DOCX 2876 kb)

Additional file 2: **Movie S1.** Animation GIF of fitting least-squares circles for Okinawa during 2012/2013. (GIF 1036 kb)

## Abbreviations

C/S/W: ILI case(s) per sentinel per week
ETM: empirical threshold method
IDWR: Infectious Disease Weekly Report
ILI: influenza-like illness
MCM: maximum curvature method
MEM: moving epidemic method
MLRM: moving logistic regression method
SMI: sentinel medical institution
SRM: segmented regression method
