# Supplementary material for: A maximum curvature method for estimating epidemic onset of seasonal influenza in Japan
Source: BMC Infect Dis. 2019 Feb 20;19:181. doi: 10.1186/s12879-019-3777-x (PMC6383251; doi:10.1186/s12879-019-3777-x)
Supplement: Supplementary file 1 — Text S1. Implementation of the empirical threshold method (ETM). Text S2. Implementation of the segmented regression method (SRM). Figure S1. Epidemic curves of weekly number of influenza cases per sentinel for the 47 prefectures in Japan from 2012-09-02 to 2018-08-26. Figure S2. Illustration of the empirical threshold method (ETM). Figure S3. Illustration of the segmented regression method (SRM). Figure S4. Box plots of epidemic characteristic parameters estimated by the ETM, SRM, and MCM for the 47 prefectures in Japan from 2012/2013 to 2017/2018. Figure S5. Epidemic onset and end estimates in Japan and three representative prefectures during influenza seasons 2012/2013–2017/2018. Figure S6. Sensitivity analysis of different n and h for epidemic onset. Figure S7. Sensitivity analysis of different n and h for epidemic end. Figure S8. Sensitivity analysis of different n and h for epidemic duration. (DOCX 2876 kb) [file 12879_2019_3777_MOESM1_ESM.docx]

Supplementary Materials for

**A maximum curvature method for estimating epidemic onset of seasonal influenza in Japan**

Jun Cai^1,2^, Bing Zhang^3^, Bo Xu^1,2^, Karen Kie Yan Chan^1,2^, Gerardo Chowell^4^, Huaiyu Tian^5^, Bing Xu^1,2*^

^1^Ministry of Education Key Laboratory for Earth System Modeling, Department of Earth System Science, Tsinghua University, Beijing 100084, China

^2^Joint Center for Global Change Studies, Beijing 100875, China

^3^School of Public Health (Shenzhen), Sun Yat-sen University, Shenzhen 518107, China

^4^School of Public Health, Georgia State University, Atlanta, Georgia 30302, USA

^5^State Key Laboratory of Remote Sensing Science, College of Global Change and Earth System Science, Beijing Normal University, Beijing 100875, China

^*^Corresponding author. Email: [bingxu@tsinghua.edu.cn](mailto:bingxu@tsinghua.edu.cn)

1. **Supplementary Text**

**Text S1** Implementation of the empirical threshold method (ETM)

Let $\left\{ y_{t}, t=1, 2, \ldots,T \right\}$ denote the weekly epidemic curve of an influenza season with $T$ weeks, where $y_{t}$ is the number of ILI cases per sentinel (referred to as intensity hereafter) reported at week $t$. Given a prespecified epidemic threshold $Y_{0}$, epidemic onset corresponds to the week $t_{o}$ in which $y_{t_{o}}$first exceeds $Y_{0}$, and epidemic end corresponds to the week $t_{e}$when $y_{t_{e}}$is decreased to be smaller than $Y_{0}$ for the first time. To avoid a false alarm of an epidemic onset, following Shoji et al.[1], we define an influenza epidemic starts to take off when $y_{t}$ has been reported to exceed $Y_{0}$ for three consecutive weeks. The algorithm of ETM is summarized as follows and is illustrated in **Fig. S2**.

Step 1. Find the peak timing $t_{p}=\underset{t=1,\ldots,T}{\text{argmax}}\{y_{t}\}$.

Step 2. Using the first half epidemic curve $\left\{ y_{t}, t=1, 2, \ldots, t_{p} \right\}$ as input, find the first week $t_{x}$ such that $y_{t}\geq Y_{0}$ for $t=t_{x},t_{x}+1, t_{x}+2$.

Step 3. If $t_{x}\geq2$, linearly interpolate the epidemic curve between $\left( t_{x}-1, y_{t_{x}-1} \right)$ and $\left( t_{x}, y_{t_{x}} \right)$ to obtain the epidemic onset $t_{o}$ such that $y_{t_{o}}=Y_{0}$; Otherwise, $t_{o}=\text{NA, }y_{t_{o}}=\mathrm{NA}$.

Step 4. Apply the same procedures as steps 2 and 3 upon the reversed second half epidemic curve $\left\{ y_{t}, t=T, T-1,\ldots,t_{p} \right\}$to find the epidemic end $t_{e}$.

Step 5. Calculate the epidemic duration as $t_{d}=t_{e}-t_{o}$.

**Text S2** Implementation of the segmented regression method (SRM)

With the same notation as in the above ETM, the procedure for implementing the SRM is summarized as follows and is illustrated in **Fig. S3**.

Step 1. Find the peak timing $t_{p}=\underset{t=1,\ldots,T}{\text{argmax}}\{y_{t}\}$.

Step 2. Fit a following piecewise linear model to capture the timing of the breakpoint in the first half epidemic curve $\left\{ y_{t}, t=1, 2, \ldots, t_{p} \right\}$, which corresponds to the epidemic onset $t_{o}$.

$$y_{t}=\beta_{0}+\beta_{1}t+\beta_{2}\left( t-t_{o} \right)^{+}+\epsilon_{t},$$

$$\epsilon_{t}\sim N(0, \sigma^{2})$$

where $\left( t-t_{o} \right)^{+}=\left( t-t_{o} \right)\times I\left( t>t_{o} \right)$ and $I$ is an indicator function. $\beta_{0}$ is the intercept, $\beta_{1}$ is the left slope, and $\beta_{2}$ is the difference-in-slope. $\epsilon_{t}$ is the normally distributed error term with mean 0 and variance $\sigma^{2}$. The R package segmented [2] is used to estimate $t_{o}$, which iteratively fits a standard linear model with an initial guess for the breakpoint, $\tilde{t_{o}}=t_{2}$.

Step 3. Linearly interpolate the epidemic curve between $(\left\lfloor t_{o} \right\rfloor, y_{\left\lfloor t_{o} \right\rfloor})$ and $(\left\lceil t_{o} \right\rceil, y_{\left\lceil t_{o} \right\rceil})$ to obtain the epidemic onset intensity $y_{t_{o}}$.

Step 4. Apply the same procedures as steps 2 and 3 upon the reversed second half epidemic curve $\left\{ y_{t}, t=T, T-1,\ldots,t_{p} \right\}$to determine the epidemic end $t_{e}$ and the epidemic ending intensity $y_{t_{e}}$.

Step 5. Calculate the epidemic duration as $t_{d}=t_{e}-t_{o}$.

1. **Supplementary Figures**


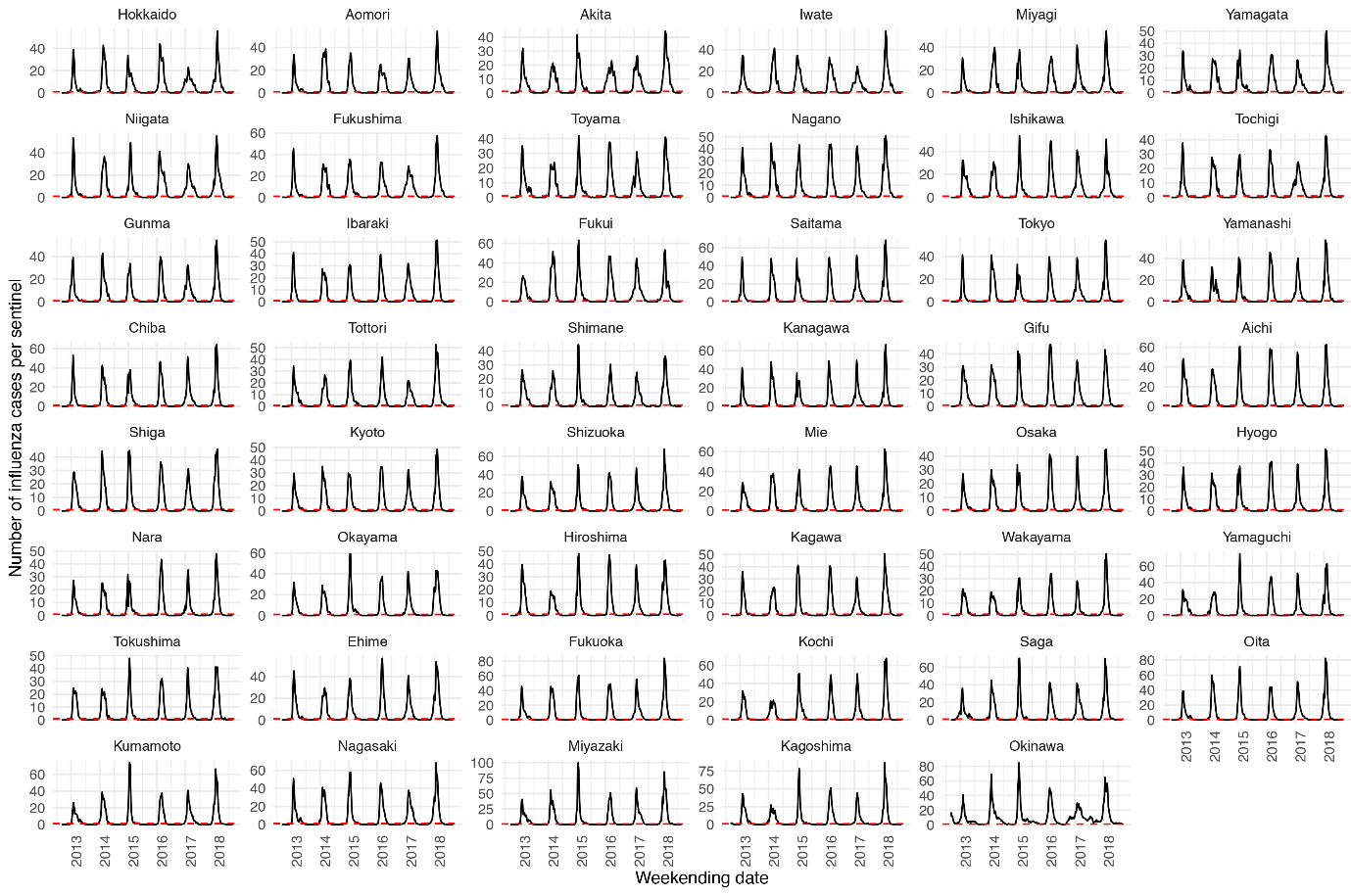


**Fig. S1** Epidemic curves of weekly number of influenza cases per sentinel for the 47 prefectures in Japan from 2012-09-02 to 2018-08-26. The red dashed horizontal line indicates the empirical epidemic threshold of 1.0 influenza-like illness case per sentinel per week. The panels are sorted by increasing latitude from bottom to top, and right to left.


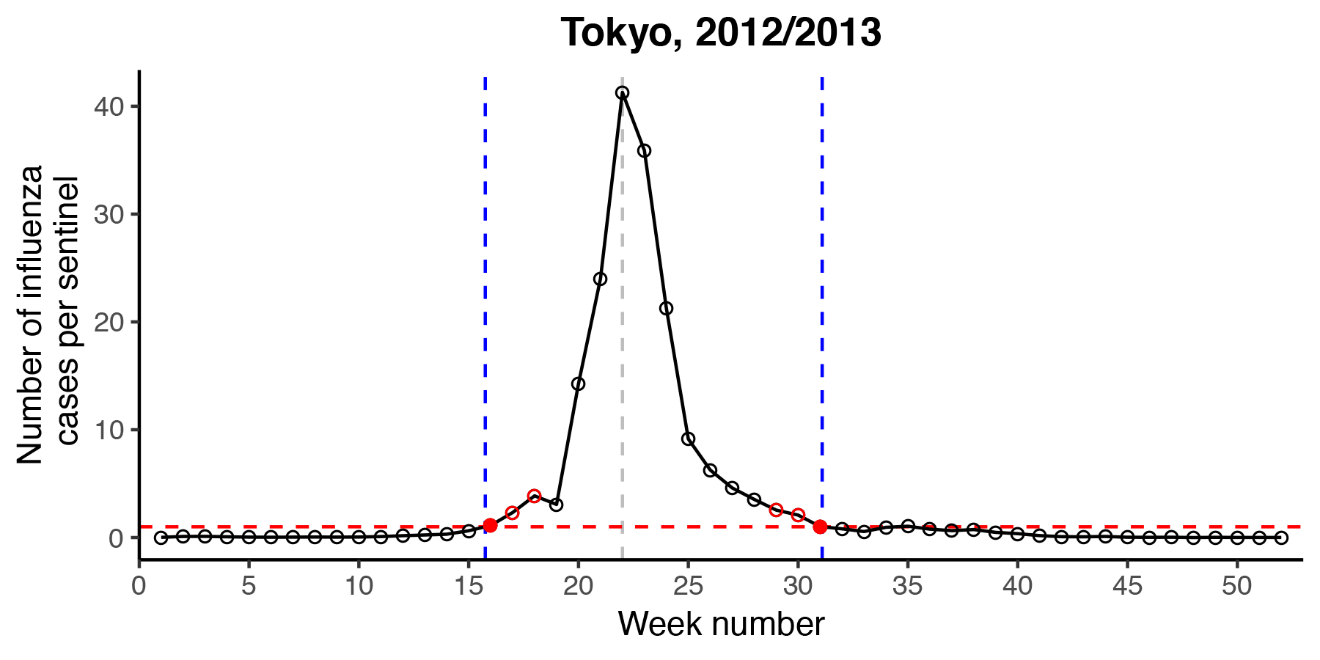


**Fig. S2** Illustration of the empirical threshold method (ETM). The gray dashed vertical line indicates the epidemic peak timing. The red dashed horizontal line indicates the empirical epidemic threshold of 1.0 influenza-like illness case per sentinel per week. The blue dashed vertical lines indicate the epidemic onset and end estimates for Tokyo during 2012/2013. The x-axis is the week number since 2012-W34.


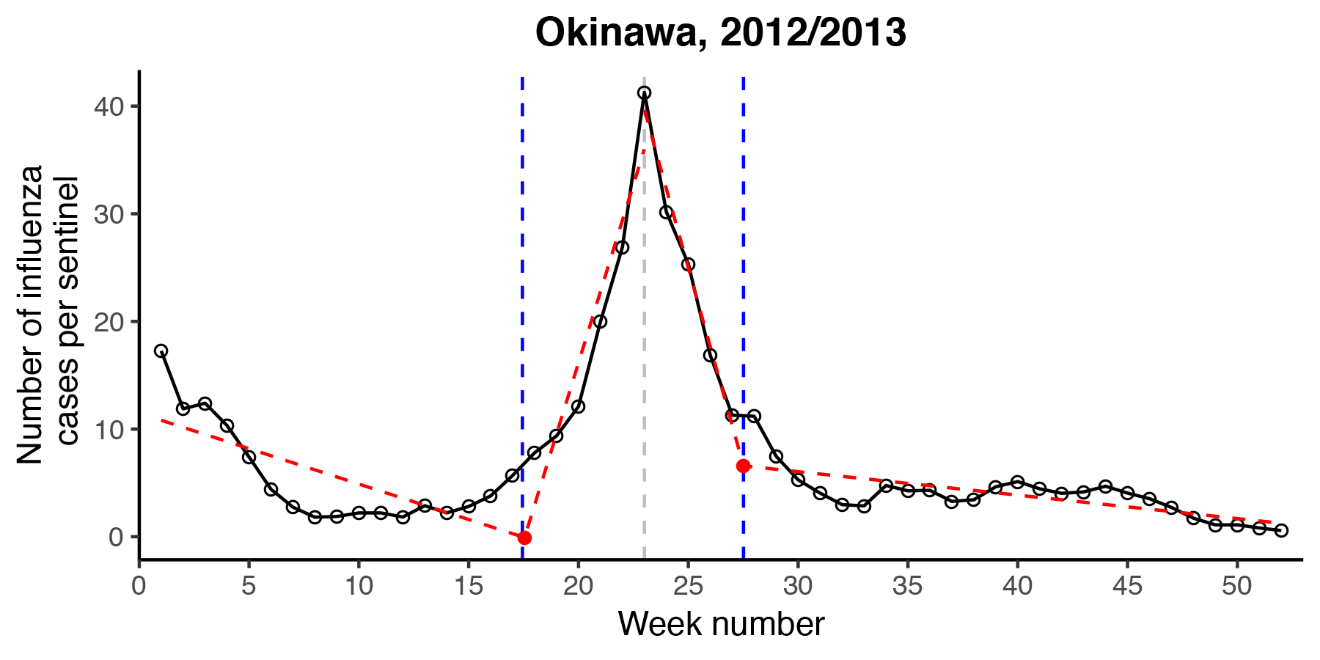


**Fig. S3** Illustration of the segmented regression method (SRM). The gray dashed vertical line indicates the epidemic peak timing. The red dashed lines are the broken-lines best fitted to the half epidemic curves. The blue dashed vertical lines indicate the epidemic onset and end estimates for Okinawa during 2012/2013. The x-axis is the week number since 2012-W34.


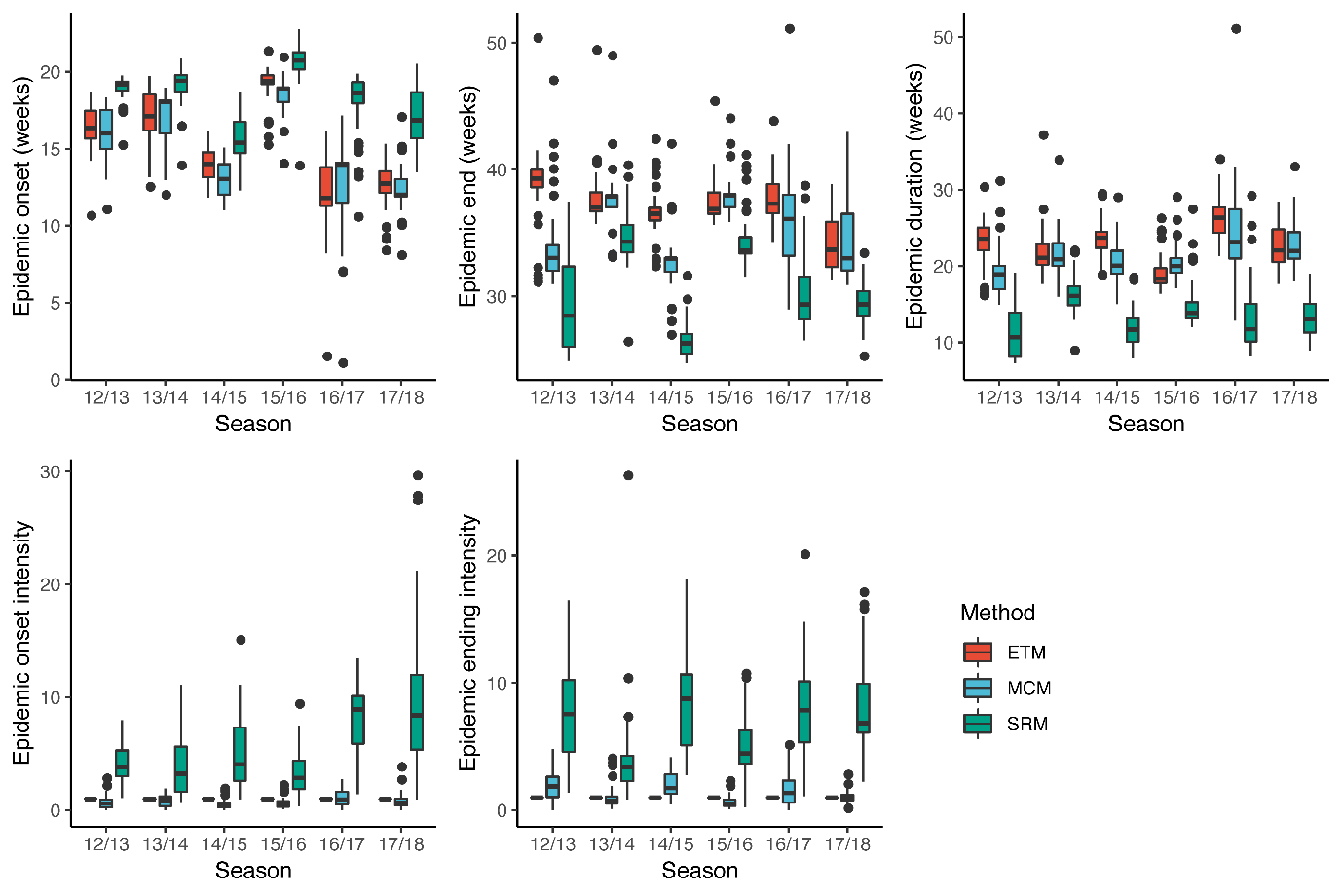


**Fig. S4** Box plots of epidemic characteristic parameters estimated by the ETM, SRM, and MCM for the 47 prefectures in Japan from 2012/2013 to 2017/2018.


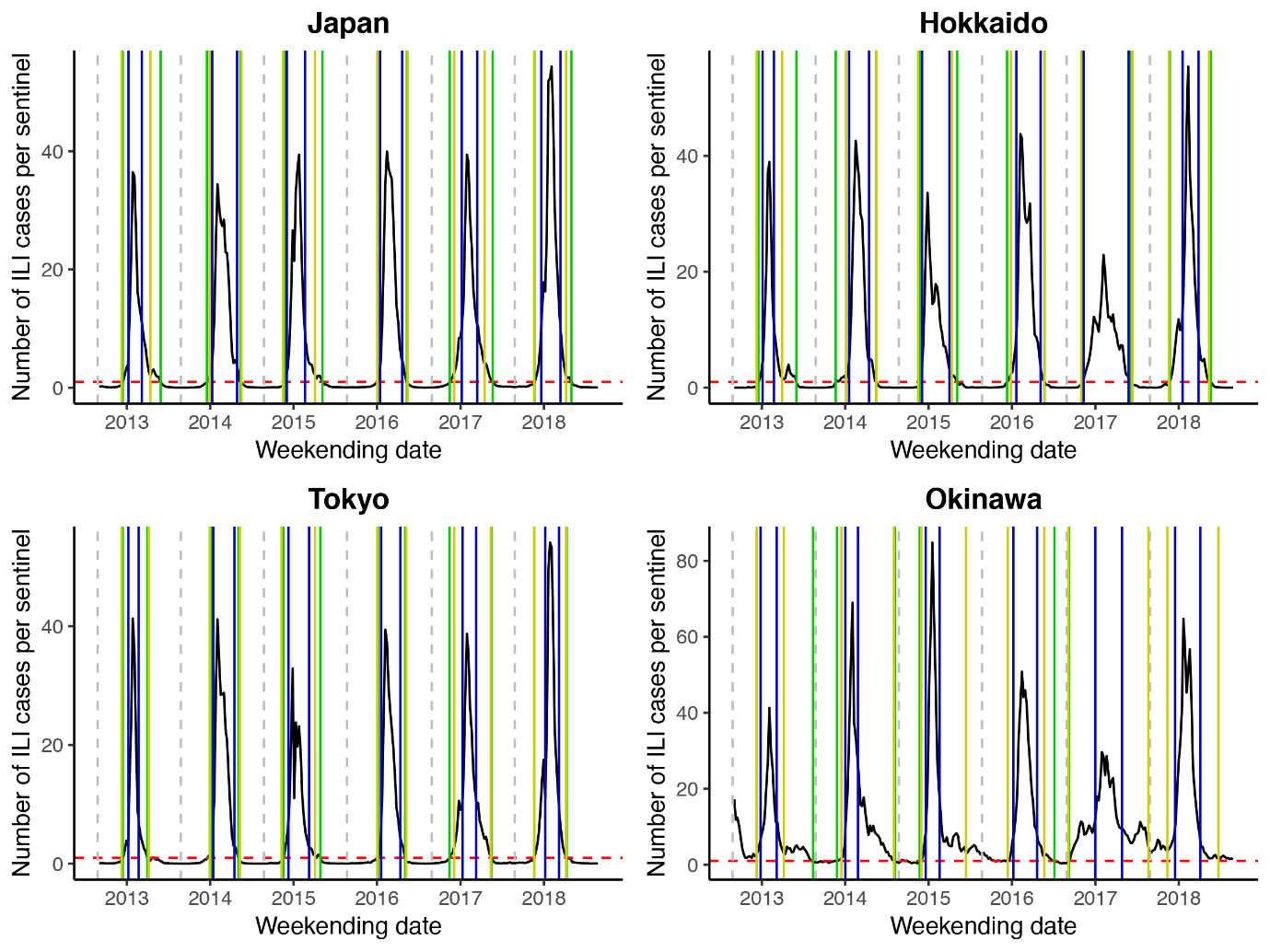


**Fig. S5** Epidemic onset and end estimates in Japan and three representative prefectures during influenza seasons 2012/2013 - 2017/2018. The gray dashed vertical lines indicate the beginning of each influenza season. The red dashed horizontal line indicates the empirical epidemic threshold of 1.0 influenza-like illness case per sentinel per week. The green, blue, and yellow vertical lines respectively represent the epidemic onset and end estimates derived by ETM, SRM, and MCM during each influenza season.


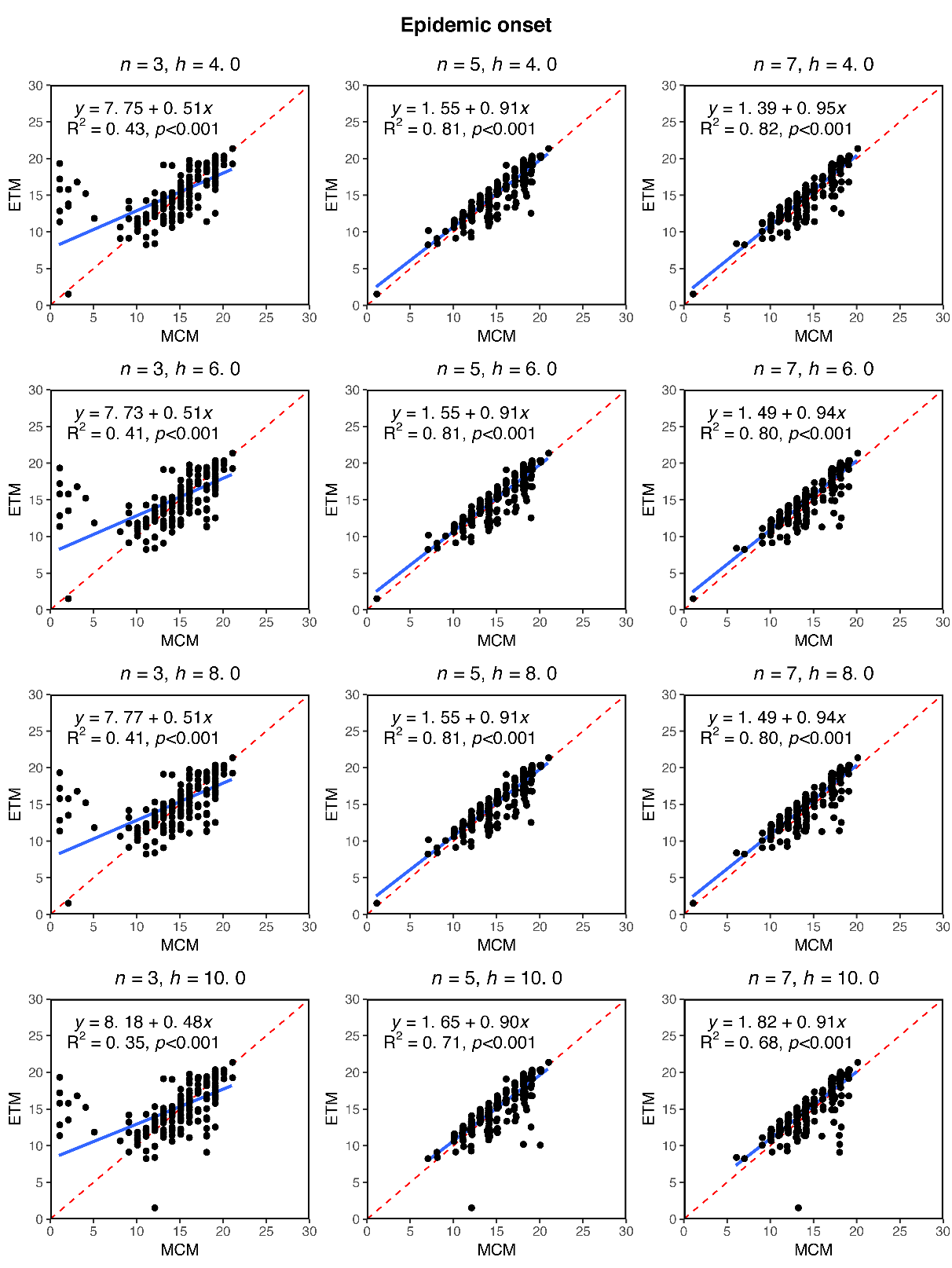


**Fig. S6** Sensitivity analysis of different $n$ and $h$ for epidemic onset. The linear regression model is used to compare epidemic onset estimates from the MCM and ETM. The regression equation, $R^{2}$ and $p$ value are annotated in each plot. The blue solid line represents the regression line, and the red dashed line displays the 1:1 line for reference in each plot.


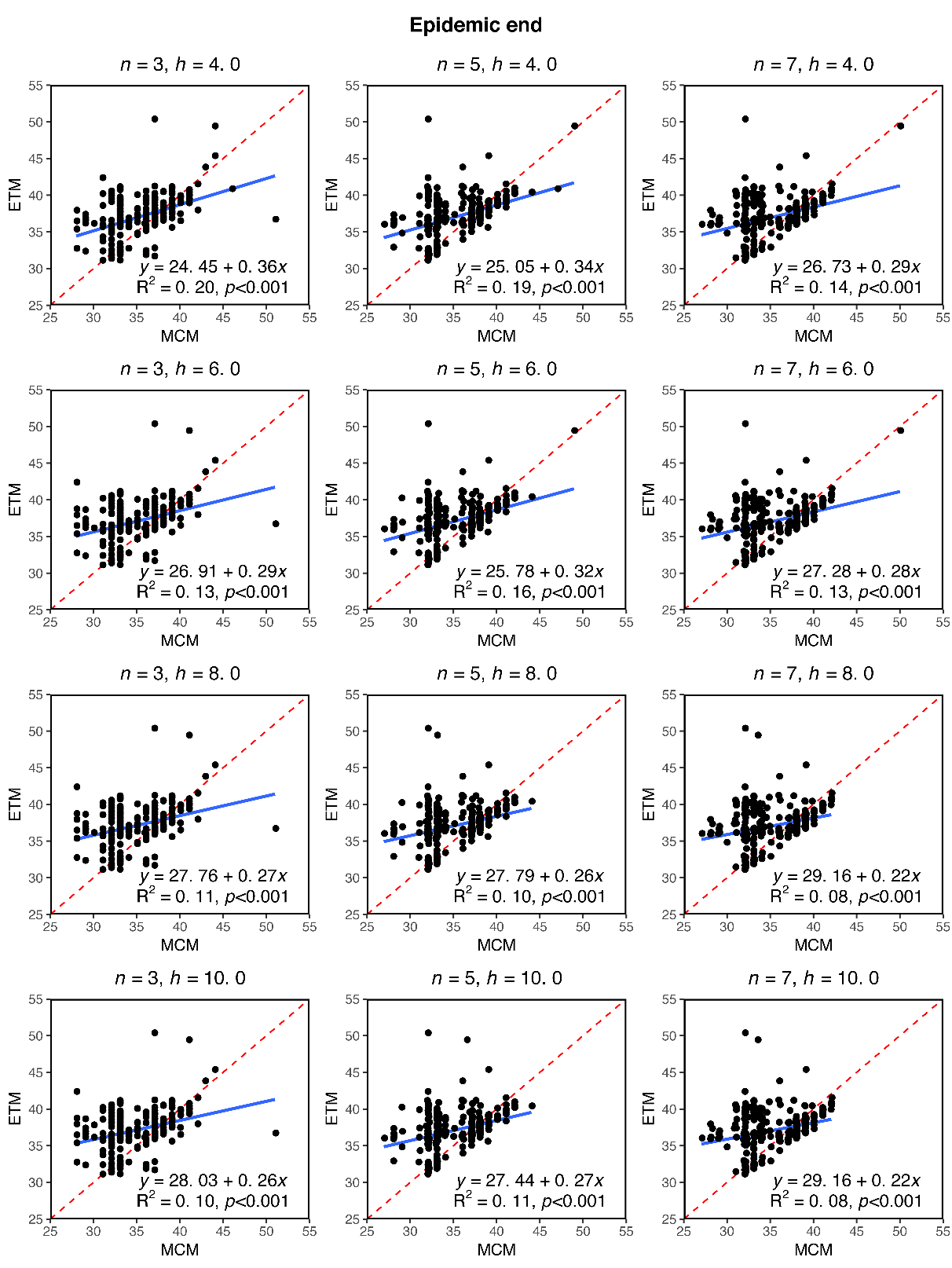


**Fig. S7** Sensitivity analysis of different $n$ and $h$ for epidemic end. The linear regression model is used to compare epidemic end estimates from the MCM and ETM. The regression equation, $R^{2}$ and $p$ value are annotated in each plot. The blue solid line represents the regression line, and the red dashed line displays the 1:1 line for reference in each plot.


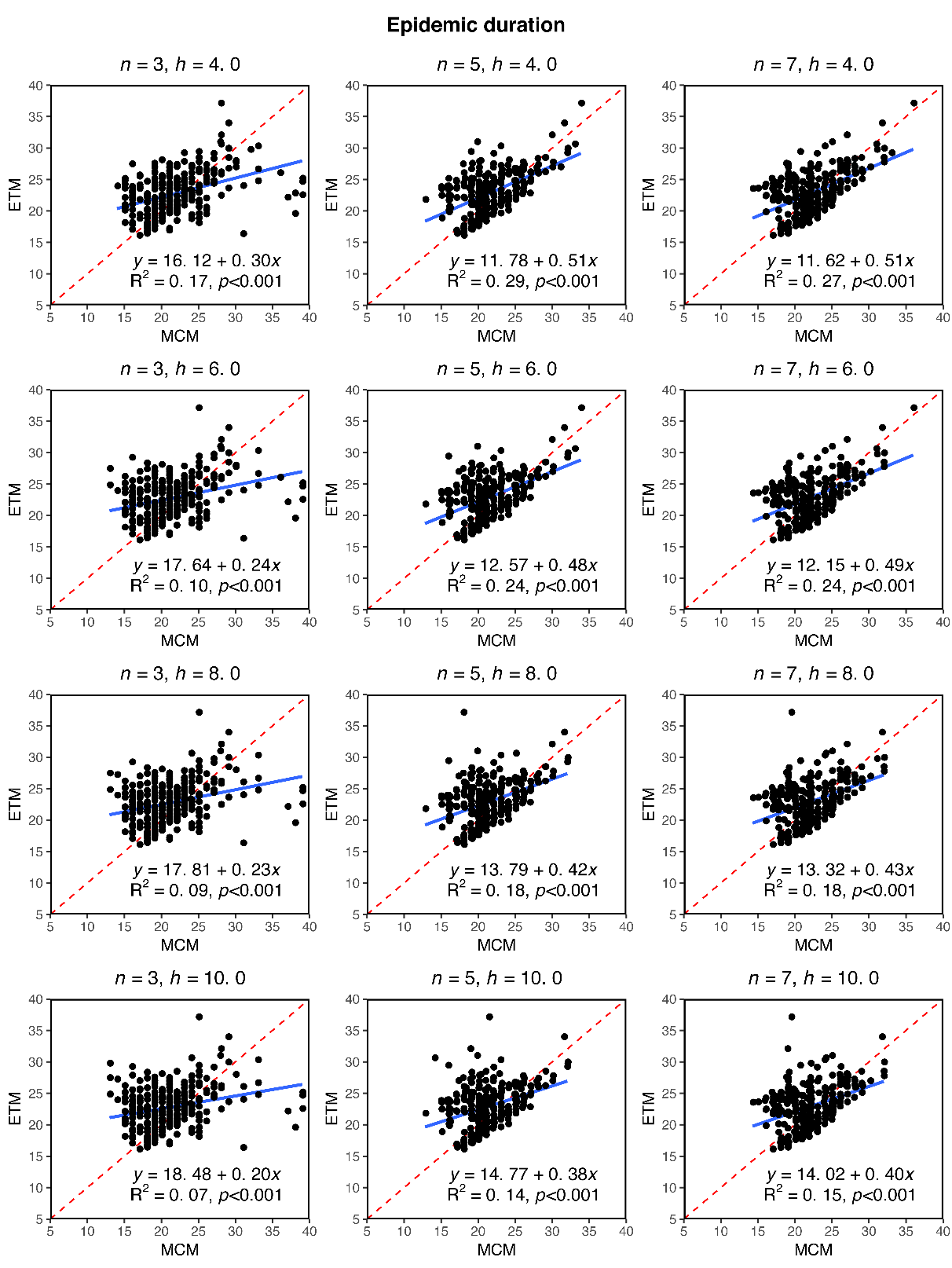


**Fig. S8** Sensitivity analysis of different $n$ and $h$ for epidemic duration. The linear regression model is used to compare epidemic duration estimates from the MCM and ETM. The regression equation, $R^{2}$ and $p$ value are annotated in each plot. The blue solid line represents the regression line, and the red dashed line displays the 1:1 line for reference in each plot.

**References**

1. Shoji M, Katayama K, Sano K. Absolute humidity as a deterministic factor affecting seasonal influenza epidemics in Japan. The Tohoku Journal of Experimental Medicine. 2011;224(4):251-6.

2. Muggeo VMR. Segmented: an R package to fit regression models with broken-line relationships. R news. 2008;8(1):20-5.
