# Supplementary figures and images for: A maximum curvature method for estimating epidemic onset of seasonal influenza in Japan
Source: BMC Infect Dis. 2019 Feb 20;19:181. doi: 10.1186/s12879-019-3777-x (PMC6383251; doi:10.1186/s12879-019-3777-x)

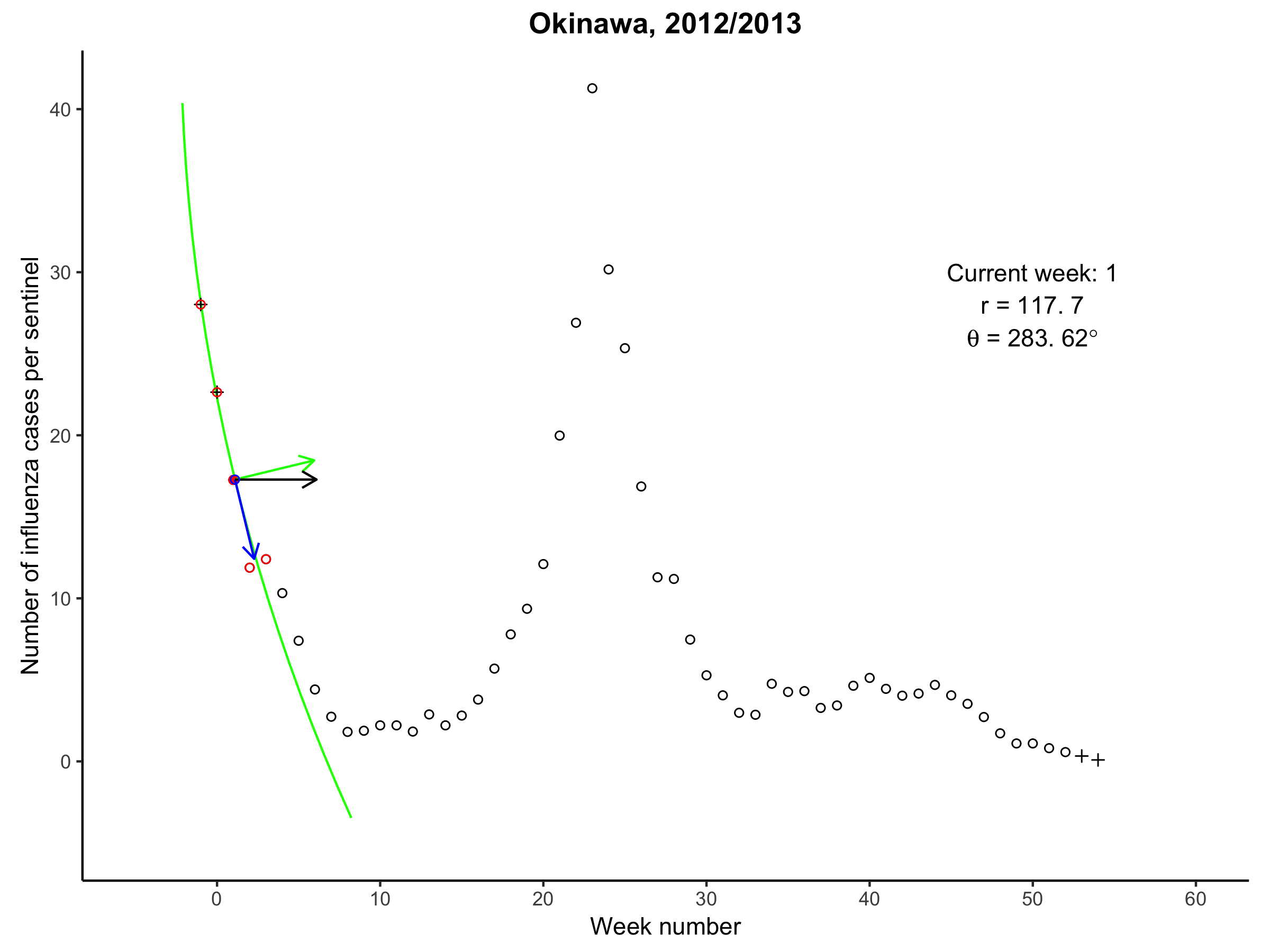

Supplement: Supplementary file 2 — Movie S1. Animation GIF of fitting least-squares circles for Okinawa during 2012/2013. (GIF 1036 kb) [file 12879_2019_3777_MOESM2_ESM.gif]
